# Transcriptome Profiling Reveals the Effects of Rootstocks on Scion Architecture in *Malus domestica* Borkh Var. ‘Harlikar’

**DOI:** 10.3390/plants14050696

**Published:** 2025-02-24

**Authors:** Bin Xie, Junhao Li, Jiangtao Zhou, Guodong Kang, Zhongwen Tang, Xiaojian Ma, Xin Li, Jing Wang, Yanzhen Zhang, Yanhui Chen, Sumiao Yang, Cungang Cheng

**Affiliations:** 1 Shijiazhuang Institute of Fruit Trees, Hebei Academy of Agriculture and Forest, Shijiazhuang 050061, China; sxauxiebin@163.com (B.X.); sxtgljh@163.com (J.L.); tt514124674@haafs.com (Z.T.); m18230188558@163.com (X.M.); 2 Key Laboratory of Mineral Nutrition and Efficient Fertilization for Deciduous Fruits, Key Laboratory of Fruit Germplasm Resources Utilization, Ministry of Agriculture and Rural Affairs, Research Institute of Pomology, Chinese Academy of Agricultural Sciences, Xingcheng 125100, China; zhoujtao@163.com (J.Z.); kgdcaas@163.com (G.K.); lixin970610@163.com (X.L.); wangjingcaas@163.com (J.W.); 17854266797@163.com (Y.Z.); cyh890324@163.com (Y.C.)

**Keywords:** apple rootstock, scion-rootstock interaction, tree architecture, endogenous hormonal regulation

## Abstract

Rootstocks largely determine the tree architecture of the grafted scions, significantly affects yield, suitability for mechanical harvesting, and planting pattern of apple orchards. It is thus important to reveal the mechanisms behind the rootstocks influence on the tree architecture of scions in apple trees. This study analyzed the grafting survival rate, the physiological parameters including plant growth, photosynthesis and nutrient accumulation in the apple variety ‘Harlikar’ with eight apple rootstocks. We also explored the mechanism of scion architecture formation using transcriptomics based on different scion/rootstock combinations. The results indicated that ‘Harlikar’ had the lowest grafting survival rate with rootstock ‘M26’, with less callus formed at the graft interface, foliage etiolation, and weak photosynthetic capacity. While ‘Harlikar’ had better affinities with ‘M9-T337’, ‘M9-Nic29’, ‘M9-Pajam2’, ‘B9’, ‘71-3-150’, ‘Qingzhen 2’, and ‘*Malus baccata*’. Among these, the highest plant height and the highest number of lateral branches were observed in ‘Harlikar’ with rootstock ‘Qingzhen 2’, they were 1.12-times and 2.0-times higher than ‘Harlikar’ with vigorous rootstock ‘*M. baccata*’, respectively. The highest accumulations of total nitrogen, total phosphorus, and total potassium in scions were observed in ‘Harlikar’/‘Qingzhen 2’, they were 2.22-times, 2.10-times, and 11.80-times higher than that in ‘Harlikar’/‘*M. baccata*’. The lowest plant height was observed in ‘Harlikar’/‘71-3-150’, only 50.47% of ‘Harlikar’/‘Qingzhen 2’ and 56.51% of ‘Harlikar’/‘*M. baccata*’, and the lowest internode length was observed in ‘Harlikar’/‘M9-Nic29’, only 60.76% of ‘Harlikar’/‘Qingzhen 2’ and 79.11% of ‘Harlikar’/‘*M. baccata*’. The transcriptome, weighted gene co-expression network and KEGG enrichment analyses revealed that, compared to ‘Harlikar’/‘*M. baccata*’, most differentially expressed genes screened from ‘Harlikar’/‘Qingzhen 2’, ‘Harlikar’/‘71-3-150’, and ‘Harlikar’/‘M9-Nic29’ were enriched in hormone signal transduction pathways. Specifically, auxin-repressed protein gene *ARP*, cytokinin synthesis related genes *CKXs* and *CYP92A6*, and brassinosteroid synthesis related gene *CYP87A3* were involved in the dwarfing of ‘Harlikar’/‘71-3-150’ and ‘Harlikar’/‘M9-Nic29’. Cytokinin synthesis related gene *ARR-A* and abscisic acid-responsive element binding factor gene *ABF* were the key to increased branching in ‘Harlikar’/‘Qingzhen 2’. In addition, acid phosphatase genes *ACPs*, and serine/threonine-protein kinase genes *PBLs* were involved in the vegetative growth of scions in ‘Harlikar’/‘Qingzhen 2’ by affecting the absorption and utilization of nutrients. These results provide theoretical guidance for cultivating high-quality ‘Harlikar’ apple trees and elucidate the molecular mechanisms regulating plant height and lateral branch formation in apple.

## 1. Introduction

Apple (*Malus domestica* Borkh.) is one of the most predominant fruit crops in the world, with the fruit providing nutritional and healthcare benefits [1]. According to the data in FAOSTAT and National Bureau of Statistics of China, China is the world’s largest apple production and consumption country, with a yield of 49.60 million tons in 2023 [2,3]. Apple production relies heavily on grafting, combining well-adapted rootstocks with high-quality scions [4]. The main aim of grafting is to reduce vegetative growth and shorten the juvenile growth period [4,5]. Rootstocks play crucial roles in various aspects of scion development, including bud outgrowth [6], lateral branch induction [7], fruit quality [8], nutrient assimilation [9,10], and adaptation to unfavorable environmental conditions [11,12]. Rootstocks affect scion architecture, especially lateral branch development and height, which decides the planting and management mode used in orchards [13]. Li et al. [14] showed that dwarfing interstocks significantly reduce the height and diameter of ‘Gala’ apple trees, with little effect on the growth of annual branches. ‘Royal Gala’ scions grafted onto ‘Royal Gala’ self-rooted stocks produce numerous vigorous primary and secondary scion shoots, whereas the same scions grafted onto ‘M9’ dwarfing rootstocks generate fewer lateral branches [15]. Chen et al. [16] reported that ‘Fuji’ apple trees grafted onto ‘*Malus baccata*’ have larger and more numerous branches than ‘Fuji’/‘M9-T337’/‘*M. baccata*’ and ‘Fuji’/‘M9-T337’. Dwarfing rootstocks have previously been shown to limit scion growth by impeding the transport of nutrients, hormones, water, and mineral salts [5,17].

Although scion architecture is known to be affected by the rootstock, the molecular mechanisms underlying these effects are poorly understood. With the continuous development of genomics-based technologies, researchers have found that rootstocks regulate the growth of grafted trees by influencing gene expression patterns in scions [16,18,19]. Liu et al. [20] reported that during grafting, there is a horizontal transfer of genetic material between rootstock and scion, resulting in a large number of differentially expressed specific genes at the graft-healing interface. Most of these genes, such as xylan endoglucosyltransferase/hydrolase genes *XTH19* and *XTH20*, invloved in cell division and healing at the rootstock interface, rather than growth regulation. Hormonal signals can control rootstock-mediated vigor by modifying gene expression in the scion part [21]. Montesinos et al. [22] noted that differences in almond tree architecture correlated with the differential expression of genes involved in hormonal and molecular responses associated with regulating apical dominance, branch formation, plant growth, cell wall formation, and nitrogen assimilation in scion. Auxin is the main regulator of establishing apical dominance and inhibiting bud outgrowth, it is synthesized in apical leaves and transported through the axis [22]. Zhang et al. reported that transcription factor gene *NF-YA10* expression in shoot tips may be part of a regulation process in the formation of branches promoting cell growth via auxin-signaling regulatory pathway [23]. Genetic changes in the biosynthesis or signaling pathways of various hormones such as zeatin riboside, brassinosteroid (BR), gibberellin (GA), abscisic acid (ABA) and jasmonic acid in scion leaves have caused a substantial impact on plant vigor and branch spreading [24]. GA-related genes are involved in regulating dwarfing traits in mango, apple and peach [15,25,26]. Apple scions with dwarfing rootstocks exhibit higher ABA levels, lower ratios of ABA to indole-3-acetic acid, and lower GA than vigorous rootstocks [27]. The transport of BR from lateral branches to the stem is crucial to lateral bud development in pear trees [18]. Xing et al. [28] reported that the leaves of scion adjacent to the top buds of spurs play a significant role in bud development and branch growth by adjusting sugar content, nitrogen (N) content, carbon (C)/N ratios, sugar metabolism-related enzyme activities and hormone contents. Moreover, Chen et al. [16] suggested that the most direct choice is to analyze leaf growth status to analyze the mechanism of rootstock-induced dwarfing, as the photosynthetic organ, the metabolism of leaves reflects the effects of grafting on tree branch architecture growth. Therefore, the analysis of the differences in gene expression patterns in leaves of ‘Harlikar’ with different rootstocks was more beneficial to revealing the mechanism of rootstock-mediated vigor by modifying gene expression in the scion part.

‘Harlikar’ is an excellent apple variety selected by Professor Kiyoshi Yokota from the natural hybrid offspring of ‘Golden Crown’ at Iwate University’s Department of Agriculture [29]. It has rapidly become one of the main varieties grown in China. Selecting suitable rootstock is the key to achieving good quality and high output for fruit trees [30]. Due to extensive planting and relatively poor orchard conditions in China, dwarfism and adaptability are the main criteria for selecting rootstocks [31]. Moreover, cultivating trees with more lateral branches and larger opening angles, architecture suitable for light capture and mechanized cropping, is particularly critical. The breeding of apple dwarfing rootstock has progressed world-wide and include the M, MAC and CG, O3, B, P, JM, Qingzhen and SH series [31]. However, rootstock/scion compatibility varies to the extent that even closely related species might not be compatible [32]. Therefore, it is necessary to select suitable rootstocks for cultivated varieties. In this study, we grafted ‘Harlikar’ onto eight rootstocks and observed the effects of the rootstocks on parameters related to tree architecture, such as plant height, branch number, internode length, leaf photosynthetic capacity, and N/P/K content. Among the eight rootstock/scion combinations, branching formation and plant height, characteristics that affect fruit cultivation, were the traits that differed most. To analyze the molecular mechanisms that underlie the modification of scion architecture by rootstocks, we used RNA sequencing to analyze gene expression patterns in leaves of ‘Harlikar’ grafted onto different dwarfing rootstocks. The findings will aid the selection and utilization of apple dwarfing rootstocks in the future and increase our understanding of the mechanisms influencing shoot growth and development in grafted trees.

## 2. Results

### 2.1. Phenotype Comparison of Apple Trees Grafted onto Different Rootstocks

**Figure 1:**
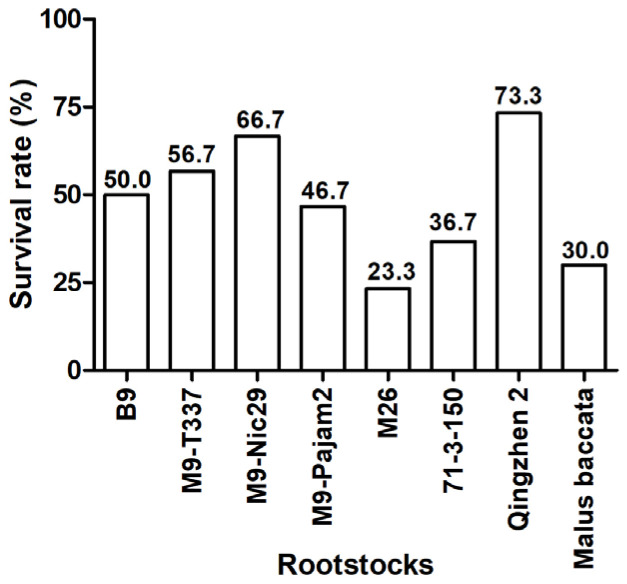
Comparison of grafting affinity among eight apple scion/rootstock combinations. Variation in the survival rate between different scion/rootstock combinations >5% were considered biologically significant.

**Figure A1:**
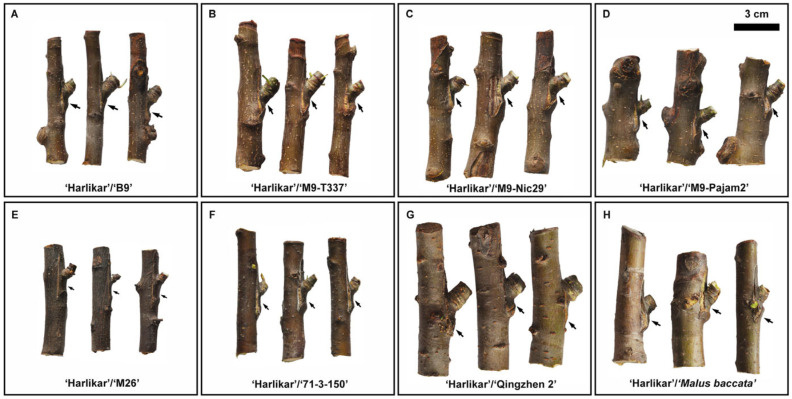
The healing status of grafted union tissue in (**A**) ‘Harlikar’/‘B9’, (**B**) ‘Harlikar’/‘M9-T337’, (**C**) ‘Harlikar’/‘M9-Nic29’, (**D**) ‘Harlikar’/‘M9-Pajam2’, (**E**) ‘Harlikar’/‘M26’, (**F**) ‘Harlikar’/‘71-3-150’, (**G**) ‘Harlikar’/‘Qingzhen 2’, and (**H**) ‘Harlikar’/‘*M. baccata*’, respectively. Scale bar = 3 cm. The arrows in the figure indicate grafting union in different apple scion/rootstock combinations.

As shown in Figure 1, the most successful grafting combination was ‘Harlikar’/‘Qingzhen 2’, which exhibited the highest survival rates, reaching 73.3%. The next most successful was ‘Harlikar’/‘M9-Nic29’, which also showed higher survival rates than the remaining scion/rootstock combinations. However, only 23.3% of the grafted ‘Harlikar’/‘M26’ trees survived. The initial wound response and callus formation at the graft interface are important manifestations of rootstock-scion compatibility. As shown in Figure A1, there were significant differences in the healing status of graft union tissue among eight apple scion/rootstock combinations. New and active calluses were produced at the junction of scion and rootstock in ‘Harlikar’/‘B9’, ‘Harlikar’/‘M9-T337’, ‘Harlikar’/‘M9-Nic29’, ‘Harlikar’/‘M9-Pajam2’, ‘Harlikar’/‘71-3-150’, ‘Harlikar’/‘Qingzhen 2’, and ‘Harlikar’/‘*M. baccata*’. However, less callus was observed in the grafted union of ‘Harlikar’/‘M26’.

**Figure 2:**
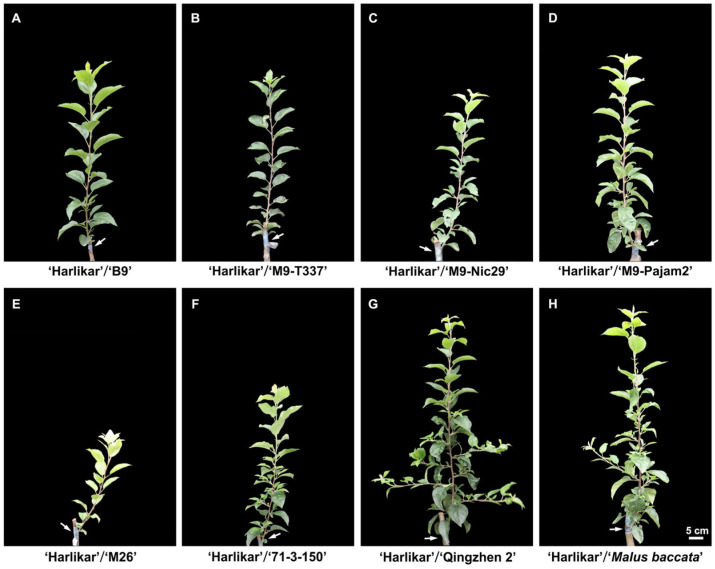
Phenotypic traits of eight apple scion/rootstock grafting combinations. ‘Harlikar’ scion architecture grafted on the (**A**) ‘B9’, (**B**) ‘M9-T337’, (**C**) ‘M9-Nic29’, (**D**) ‘M9-Pajam2’, (**E**) ‘M26’, (**F**) ‘71-3-150’, (**G**) ‘Qingzhen 2’, and (**H**) ‘*Malus baccata*’. Scale bar = 5 cm. The white arrows in the figure indicate grafting union in different apple scion/rootstock combinations.

**Figure 3:**
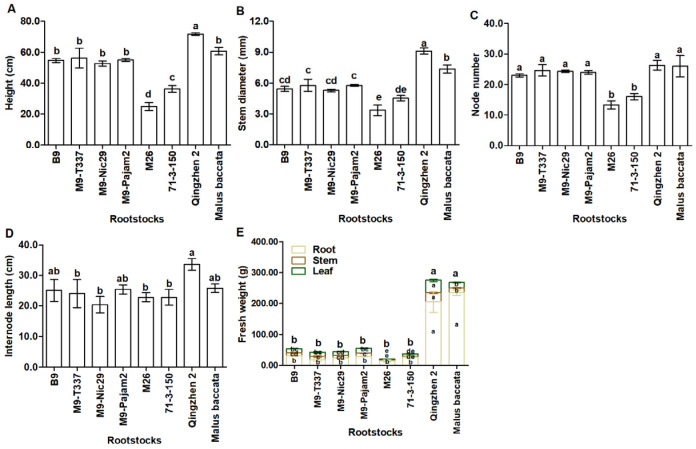
Comparison of plant vegetative growth parameters among grafting combinations of ‘Harlikar’ apple scions with eight different rootstocks. The effect of rootstocks on (**A**) plant height, (**B**) scion stem diameter, (**C**) scion node number, and (**D**) scion internode length of apple trees. (**E**) Differences in the fresh weights of leaves, stems, and roots among eight scion/rootstock combinations. Data were shown as means ± SE (*n* = 3), and different lowercase letters above the bars indicate significant differences among different apple scion/rootstock combinations (*p* < 0.05).

As shown in Figure 2 and Table A1, the rootstocks influenced vigor of scions. ‘Qingzhen 2’ rootstock produced the tallest ‘Harlikar’ trees with thickest stem, its height and stem diameter was 1.12-fold and 1.25-fold higher than that of ‘Harlikar’/‘*M. baccata*’. While the plant heights and stem diameters in ‘Harlikar’/‘M26’ were the lowest, only 38.80% and 45.93% of ‘Harlikar’/‘*M. baccata*’, followed by ‘Harlikar’/‘71-3-150’, only 56.51% and 61.92% of ‘Harlikar’/‘*M. baccata*’. There was no significant difference in the plant height among ‘Harlikar’/‘B9’, ‘Harlikar’/‘M9-T337’, ‘Harlikar’/‘M9-Nic29’, ‘Harlikar’/‘M9-Pajam2’, and ‘Harlikar’/‘*M. baccata*’ (Figure 3A,B, Table A1). ‘Harlikar’/‘M26’ and ‘Harlikar’/‘71-3-150’ produced the fewest nodes, but there was no significant difference in the node number among the remaining six apple scion/rootstock combinations (Figure 3C, Table A1). As shown in Figure 2 and Table A1, the highest number of branches was observed in ‘Harlikar’/‘Qingzhen 2’, and was 2.0-fold higher than ‘Harlikar’/‘*M. baccata*’, and no obvious branch was observed in stems of ‘Harlikar’/‘B9’, ‘Harlikar’/‘M9-T337’, ‘Harlikar’/‘M9-Nic29’, ‘Harlikar’/‘M9-Pajam2’, and ‘Harlikar’/‘M26’. The highest internode length was observed in ‘Harlikar’/‘Qingzhen 2’, and the lowest was observed in ‘Harlikar’/‘M9-Nic29’, the internode length of ‘Harlikar’/‘Qingzhen 2’ was 1.65-fold higher than that of ‘Harlikar’/‘M9-Nic29’. There was no significant difference among ‘Harlikar’/‘B9’, ‘Harlikar’/‘M9-T337’, ‘Harlikar’/‘M9-Nic29’, ‘Harlikar’/‘M9-Pajam2’, ‘Harlikar’/‘M26’, ‘Harlikar’/‘71-3-150’ and ‘Harlikar’/‘*M. baccata*’ (Figure 3D, Table A1). As shown in Figure 3E and Table A2, the fresh weights of leaves and stems in ‘Harlikar’/‘Qingzhen 2’ were the highest, followed by ‘Harlikar’/‘*M. baccata*’, the fresh weights of roots in ‘Harlikar’/‘*M. baccata*’ were the highest, followed by ‘Harlikar’/‘Qingzhen 2’, while the fresh weights of leaves, stems and roots in ‘Harlikar’/‘M26’ was the lowest among the eight combinations.

**Figure 4:**
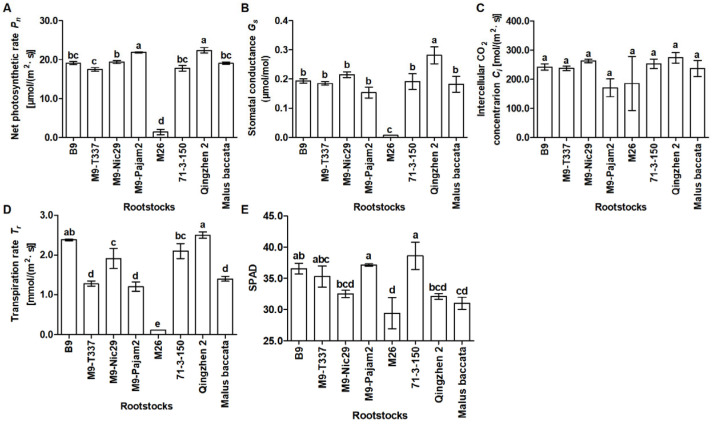
Comparison of photosynthetic parameters of scion leaves in grafting combinations of ‘Harlikar’ apple scions with eight different rootstocks. The effects of rootstock on (**A**) the net photosynthetic rate (*P_n_*), (**B**) stomatal conductance, (**C**) intercellular CO_2_ concentration (*C_i_*), (**D**) transpiration rate (*T_r_*), and (**E**) SPAD of leaves in the grafted trees. Data were shown as means ± SE (*n* = 3), and different lowercase letters above the bars indicate significant differences among different apple scion/rootstock combinations (*p* < 0.05).

According to Figure 4A,B,D and Table A3, the *P_n_*, *G_s_,* and *T_r_* of leaves in ‘Harlikar’/‘M26’ was the lowest among eight apple scion/rootstock combinations. The *P_n_* and *G_s_* of leaves in ‘Harlikar’/‘M9-Pajam2’ and ‘Harlikar’/‘Qingzhen 2’ was significantly higher than in the remaining six apple scion/rootstock combinations (Figure 4A,B, Table A3), and the *G_s_* of leaves in ‘Harlikar’/‘Qingzhen 2’ was significantly higher than in the remaining seven apple scion/rootstock combinations (Figure 4B, Table A3). There was no significant difference in the *C_i_* among eight apple scion/rootstock combinations (Figure 4C, Table A3). As shown in Figure 4E and Table A3, the lowest value of SPAD was measured in ‘Harlikar’/‘M26’, only 29.39. And the highest value was measured in ‘Harlikar’/‘71-3-150’, and was 1.31-fold higher than ‘Harlikar’/‘M26’, there was no significant difference among ‘Harlikar’/‘71-3-150’, ‘Harlikar’/‘M9-Pajam2’, ‘Harlikar’/‘B9’, and ‘Harlikar’/‘M9-T337’.

### 2.2. Effects of Different Apple Rootstocks on Nutrient Uptake and Utilization in ‘Harlikar’ Apple Trees

**Figure 5:**
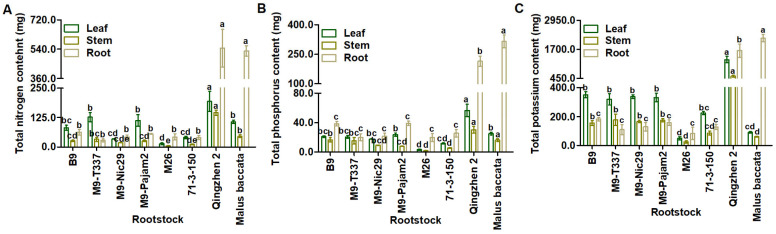
Comparison of plant nutrient accumulation among grafting combinations of ‘Harlikar’ apple scions with eight different rootstocks. Differences in the (**A**) total N, (**B**) total P, and (**C**) total K contents of leaves, stems, and roots from the grafted trees. Data were shown as means ± SE (*n* = 3), and different lowercase letters above the bars indicate significant differences among different apple scion/rootstock combinations (*p* < 0.05).

The total N content of roots in ‘Harlikar’/‘Qingzhen 2’ and ‘Harlikar’/‘*M. baccata*’ was significantly higher than that in all of the other apple scion/rootstock combinations. And the total N content of stems and leaves in ‘Harlikar’/‘Qingzhen 2’ was highest, and was 2.22-times higher than that in ‘Harlikar’/‘*M. baccata*’. The lowest total N content of stems was measured in ‘Harlikar’/‘M26’. There was no significant difference in the total N content of leaves among the combinations of ‘Harlikar’ with ‘*M. baccata*’, ‘B9’, ‘M9-T337’, and ‘M9-Pajam2’, and in the total content of roots among the combinations of ‘Harlikar’ with ‘B9’, ‘M9-T337’, ‘M9-Nic29’, ‘M9-Pajam2’, ‘M26’ and ‘71-3-150’ (Figure 5A, Table A4).

The total P content of roots in ‘Harlikar’/‘Qingzhen 2’ and ‘Harlikar’/‘*M. baccata*’ was substantially higher than that in the remaining six apple scion/rootstock combinations, and there was no significant difference in the total P content of leaves among the combinations of ‘Harlikar’ with ‘*M. baccata*’, ‘B9’, ‘M9-T337’, ‘M9-Nic29’, and ‘M9-Pajam2’. The total P content of scions (stems and leaves) was 2.10-times higher than that in ‘Harlikar’/‘*M. baccata*’ (Figure 5B, Table A5).

The total K content of ‘Harlikar’/‘*M. baccata*’ roots was highest, while that of its stems was lowest, and the total K content of its leaves was lower than all except ‘Harlikar’/‘M26’ and ‘Harlikar’/‘71-3-150’. The highest accumulation of total K in scions (stems and leaves) was observed in ‘Harlikar’/‘Qingzhen 2’, theyit was 11.80-times higher than that in ‘Harlikar’/‘*M. baccata*’ (Figure 5C, Table A6).

### 2.3. Transcriptome Analysis of Leaves from ‘Harlikar’ Apple Trees Grafted onto Different Rootstocks

In order to further elucidate the molecular mechanisms of rootstock affecting height and branch morphology of apple scion, we then compared and analyzed the difference of gene expression patterns in leaves of different scion/rootstock combinations. In this study, the combination of ‘Harlikar’ with ‘*M. baccata*’, a traditional vigorous apple rootstock commonly used in China, was set as a control. ‘Harlikar’/‘Qingzhen 2’ with highest plant height and the largest number of lateral branches, ‘Harlikar’/‘71-3-150’ with good affinity and lowest plant height, and ‘Harlikar’/‘M9-Nic29’ with good affinity and lowest internode length were set as treatments.

**Figure 6:**
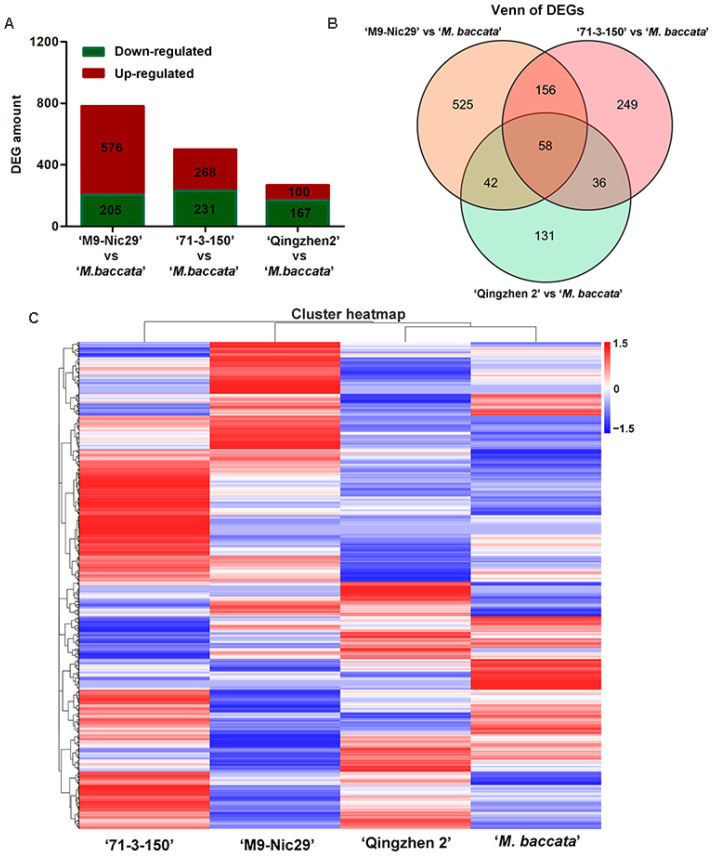
Transcriptome changes in ‘Harlikar’ apple scions grafted onto four different rootstocks. (**A**) The number of differentially expressed genes (DEGs) in comparisons of ‘Harlikar’/‘M9-Nic29’ vs. ‘Harlikar’/‘*M. baccata*’, ‘Harlikar’/‘71-3-150’ vs. ‘Harlikar’/‘*M. baccata*’, and ‘Harlikar’/‘Qingzhen 2’ vs. ‘Harlikar’/‘*M. baccata*’. (**B**) Venn diagram showing common and overlapped DEGs among the different comparison groups. (**C**) Hierarchical clustering of the global transcriptome of the four scion/rootstock combinations. Each column represents a sample, each row represents a gene. The color represents the expression value of the gene after standardized treatment in each sample. Red indicates higher gene expression, and blue indicates lower gene expression.

Transcriptome sequencing generated an average of 43,503,648 reads and 42,702,312 clean reads per sample (Table A7). There were 1547 differentially expressed genes (DEGs) identified from the comparisons of ‘Harlikar’/‘M9-Nic29’ vs. ‘Harlikar’/‘*M. baccata*’, ‘Harlikar’/‘71-3-150’ vs. ‘Harlikar’/‘*M. baccata*’, and ‘Harlikar’/‘Qingzhen 2’ vs. ‘Harlikar’/‘*M. baccata*’ (Figure 6A).

As shown in Figure 6, compared with ‘Harlikar’/‘*M. baccata*’, 781, 499, and 267 DEGs were screened in the leaves of ‘Harlikar’ combined with ‘M9-Nic29’, ‘71-3-150’, and ‘Qingzhen 2’, respectively. Overall, 73.8% of the DEGs from scions grafted onto ‘M9-Nic29’ were up-regulated, more DEGs were up-regulated than down-regulated using ‘71-3-150’ rootstock, and 62.5% of DEGs were down-regulated in ‘Harlikar’/‘Qingzhen 2’. Compared with ‘Qingzhen 2’, ‘M9-Nic29’ and ‘71-3-150’ had stronger and more unique effects on the transcriptome of ‘Harlikar’.

**Figure 7:**
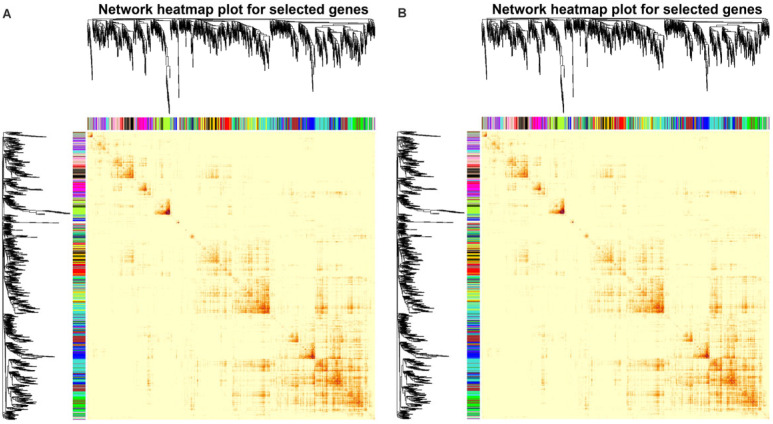
Weighted gene co-expression network analysis of DEGs, growth phenotypes (**A**), and nutrient content (**B**) in ‘Harlikar’ apple scions grafted onto ‘M9-Nic29’, ‘71-3-150’, and ‘Qingzhen 2’ rootstocks. The top is hierarchical clustering tree of genes, and the bottom were clustering heatmap of gene modules. Each branch represents a gene, and each tree represents a gene module. The redder the color of each point in the heatmap, the stronger the interaction between the two genes corresponding to the row and column.

**Figure A2:**
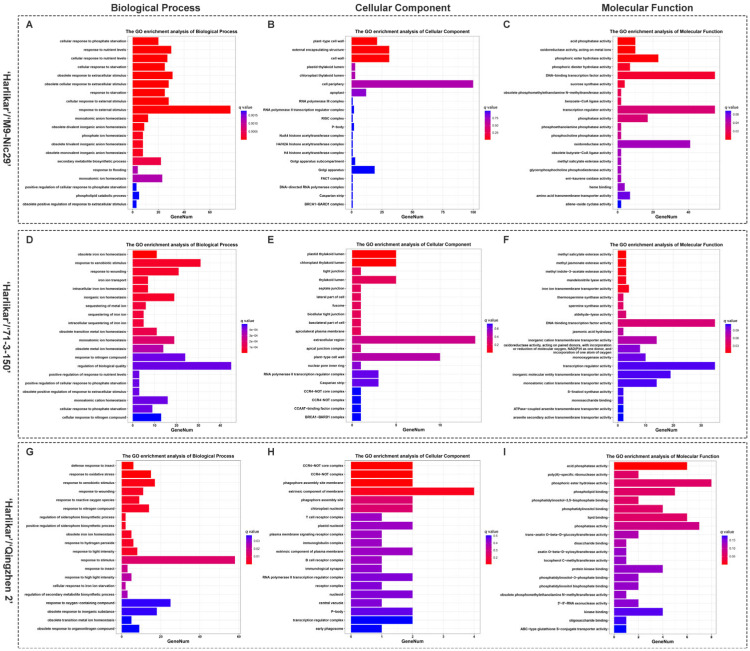
GO enrichment of differentially expressed genes (DEGs) in combinations with ‘Harlikar’ as the scion. Molecular function terms for DEGs in combinations with (**A**) ‘M9-Nic29’, (**D**) ‘71-3-150’, and (**G**) ‘Qingzhen 2’ as the rootstock, respectively. Cellular component for DEGs in combinations with (**B**) ‘M9-Nic29’, (**E**) ‘71-3-150’, and (**H**) ‘Qingzhen 2’ as the rootstock, respectively. Molecular function for DEGs in combinations with (**C**) ‘M9-Nic29’, (**F**) ‘71-3-150’, and (**I**) ‘Qingzhen 2’ as the rootstock, respectively. The smaller the *q*-value, and the more significant the enrichment.

**Figure A3:**
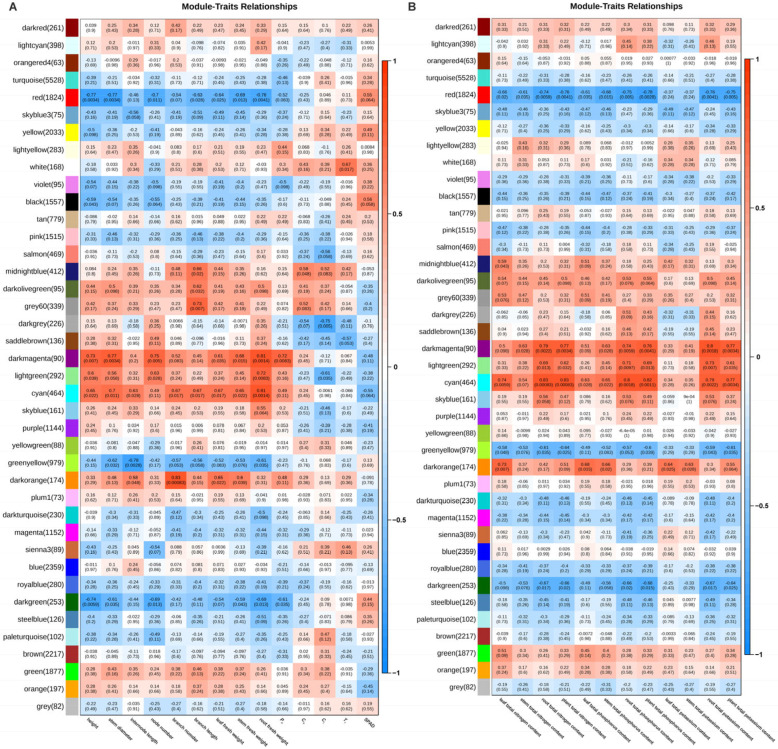
Correlation between modules of DEGs and traits including growth phenotypes (**A**) and nutrient content (**B**) of four scion/rootstock combinations.

A weighted gene co-expression network analysis (WGCNA) was used to identify genes highly correlated with plant height, branch number and nutrient content (Figure 7). A total of 10 modules, black, cyan, darkgreen, darkmagenta, darkolivegreen, darkorange, greenyellow, lightgreen, midnightblue, and red, were screened out. The height and nutrient content were negatively correlated with the MEred and MEdarkgreen modules but positively correlated with the MEdarkmagenta, MElightgreen, MEcyan, and MEdarkorange modules. Moreover, branch number was positively correlated with the MEcyan and MEdarkorange modules (Figure A3). The DEGs in the MEred module were enriched in transferase activity, transferring phosphorus-containing groups, and serine/threonine kinase activity. Those in the MEcyan module were enriched in molecular transducer activity, while the DEGs in the MEdarkmagenta module were enriched in photosynthesis (Figure A2).

**Figure 8:**
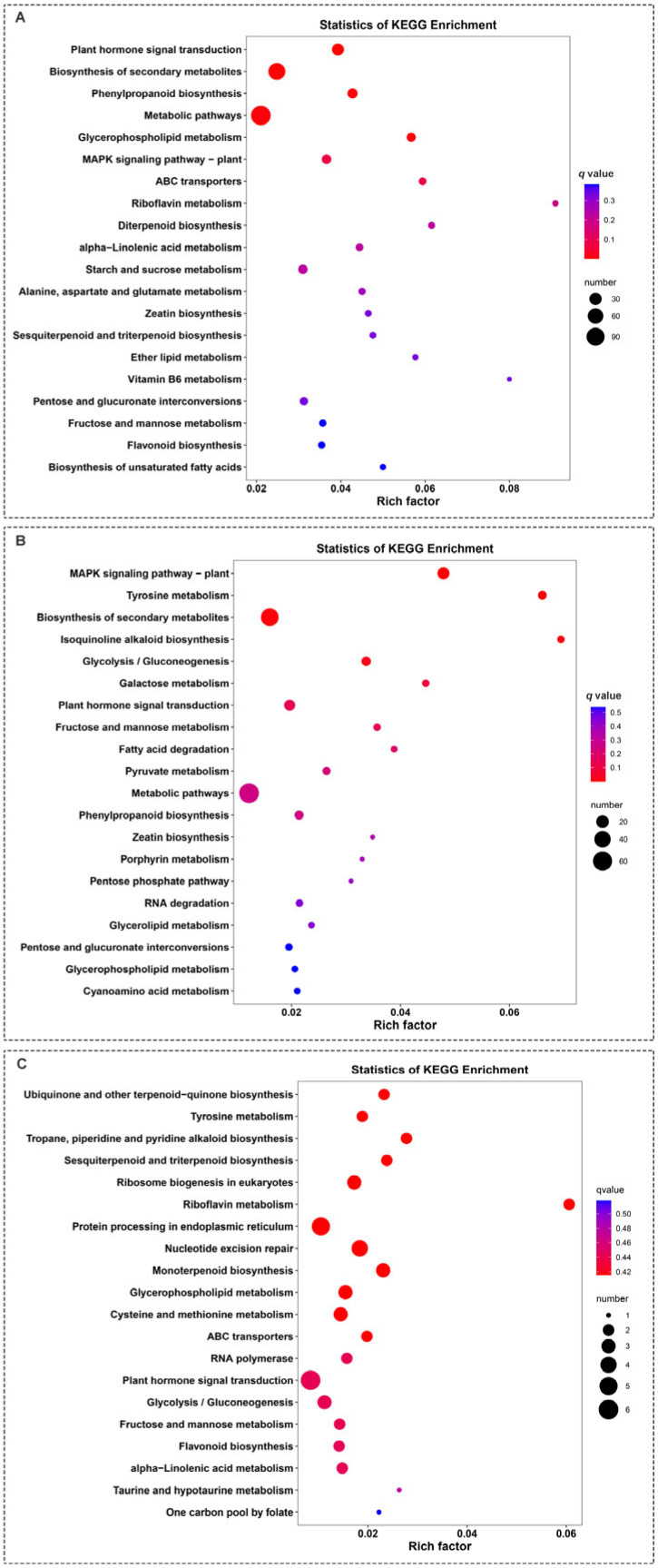
KEGG enrichment of DEGs in ‘Harlikar’ apple grafted onto three different rootstocks. The DEGs in comparisons of (**A**) ‘Harlikar’/‘M9-Nic29’ vs. ‘Harlikar’/‘*M. baccata*’, (**B**) ‘Harlikar’/‘71-3-150’ vs. ‘Harlikar’/‘*M. baccata*’, and (**C**) ‘Harlikar’/‘Qingzhen 2’ vs. ‘Harlikar’/‘*M. baccata*’. Rich factor refers to the ratio of the number of DEGs enriched in the pathway to the number of annotated genes. The greater the Rich factor, the greater the degree of enrichment, the smaller the *q*-value, and the more significant the enrichment.

A Kyoto Encyclopedia of Genes and Genomes (KEGG) pathway enrichment analysis revealed that the DEGs screened from the leaves of ‘Harlikar’ grafted onto ‘M9-Nic29’ and ‘71-3-150’ were primarily enriched in metabolic pathways and the biosynthesis of secondary metabolites. Riboflavin metabolism was the most enriched pathway in comparisons of ‘Harlikar’/‘M9-Nic29’ vs. ‘Harlikar’/‘*M. baccata*’ and ‘Harlikar’/‘Qingzhen 2’ vs. ‘Harlikar’/‘*M. baccata*’. Most of the DEGs among the comparisons of ‘Harlikar’/‘M9-Nic29’ vs. ‘Harlikar’/‘*M. baccata*’, ‘Harlikar’/‘71-3-150’ vs. ‘Harlikar’/‘*M. baccata*’, and ‘Harlikar’/‘Qingzhen 2’ vs. ‘Harlikar’/‘*M. baccata*’ were enriched in plant hormone biosynthesis and signal transduction pathways (Figure 8).

**Figure 9:**
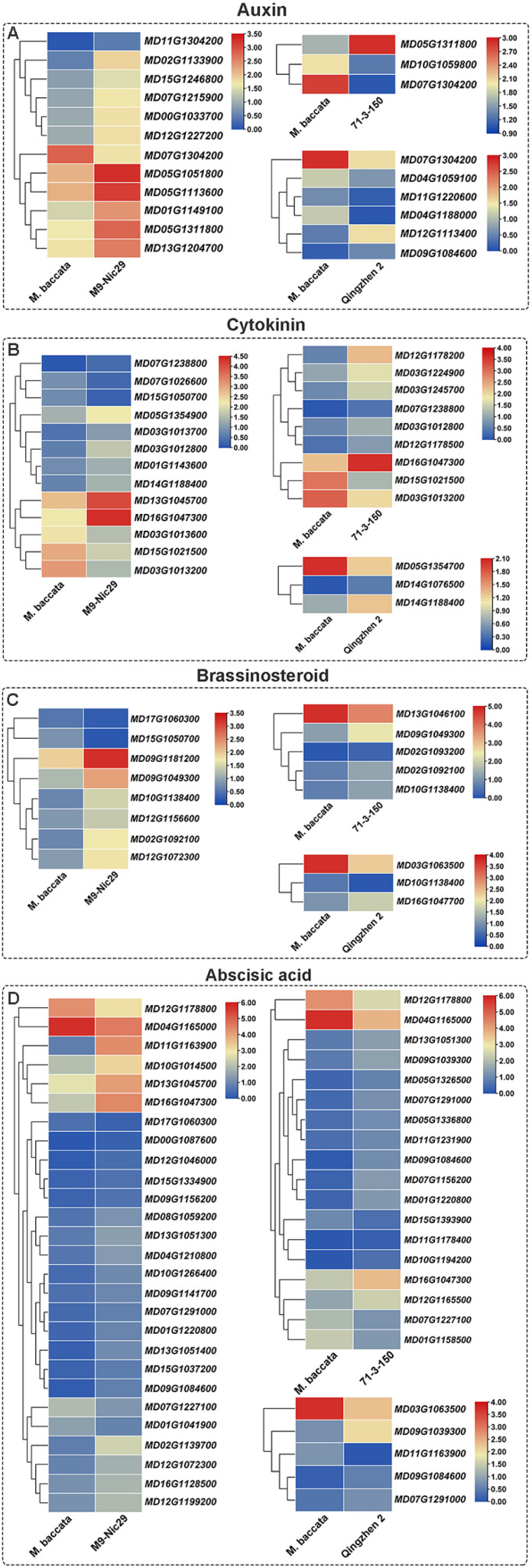
Heat map of differentially-expressed genes (DEGs) related to the signaling pathways of seven hormones in ‘Harlikar’ grafted onto three different rootstocks. The DEGs were related to (**A**) auxin, (**B**) cytokinin, (**C**) brassinosteroid, and (**D**) abscisic acid. The color scale on each heatmap shows log_2_ FPKM.

Overall, 146 DEGs related to the biosynthesis and signal transduction of six plant hormones were screened (Figure 9). Of these ‘Harlikar’/‘Qingzhen 2’, ‘Harlikar’/‘M9-Nic29’ and ‘Harlikar’/‘71-3-150’ had the most DEGs in common, and these genes exhibited the same expression trends. The unique DEGs related to auxin biosynthesis and signal transduction were up-regulated in ‘Harlikar’/‘M9-Nic29’; the auxin-responsive protein gene *MD12G1113400* (*SRUR*) and allene oxide cyclase coded gene *MD09G1084600* (*AOC*) were up-regulated in ‘Harlikar’/‘Qingzhen 2’; and the auxin-repressed protein gene *MD05G1311800* (*ARP*) was up-regulated in both ‘Harlikar’/‘M9-Nic29’ and ‘Harlikar’/‘71-3-150’. In contrast, the auxin-responsive protein gene *MD10G1059800* (*SRUR21*) and auxin canalization gene *MD07G1304200* (*VAN3*) were down-regulated in ‘Harlikar’/‘71-3-150’ (Figure 9A).

**Figure A4:**
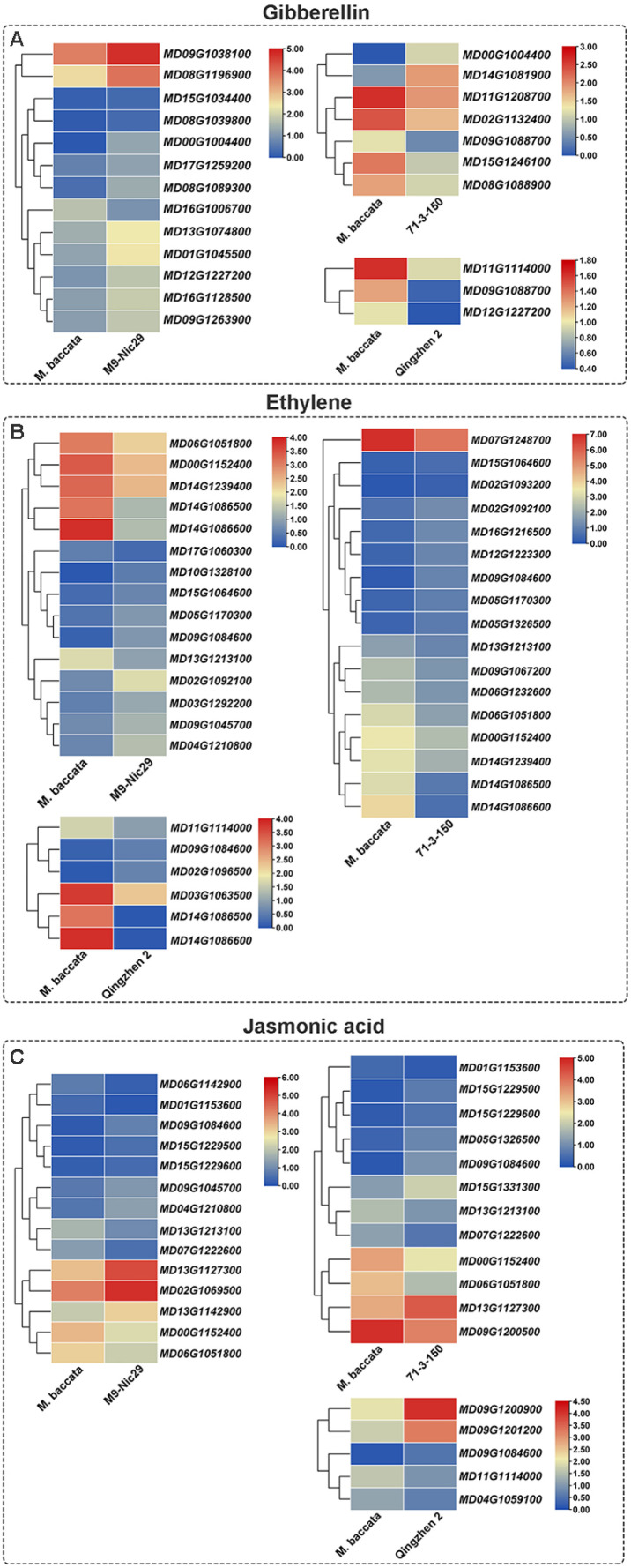
Heat map of differentially-expressed genes (DEGs) related to the signaling pathways of seven hormones in ‘Harlikar’ grafted onto three different rootstocks. The DEGs were related to (**A**) gibberelin, (**B**) ethylene, and (**C**) jasmonic acid. The color scale on each heatmap shows log_2_ FPKM.

All DEGs involved in the biosynthesis and signal transduction of GA in ‘Harlikar’/‘Qingzhen 2’ were down-regulated, and five, including GA regulated protein genes (*GASAs*; *MD15G1246100*, *MD02G1132400*, and *MD08G1088900*) were down-regulated in ‘Harlikar’/‘71-3-150’, while most in ‘Harlikar’/‘M9-Nic29’ were up-regulated, including the significantly up-regulated GA receptor gene *MD12G1227200* (*GID*). Moreover, the two-component response regulator ARR-A family member *MD14G1188400* (*ARR-A*) was up-regulated in ‘Harlikar’/‘Qingzhen 2’ (Figure A4A).

CK dehydrogenase genes such as *MD15G1021500* and *MD07G1026600* (*CKXs*) were down-regulated in ‘Harlikar’/‘71-3-150’ and ‘Harlikar’/‘M9-Nic29’, as was the CK trans-hydroxylase gene *MD15G1050700* (*CYP735A*) in ‘Harlikar’/‘M9-Nic29’. *MD03G1012800*, *MD03G1012800*, and *MD03G1013700*, encoding peroxidase, were up-regulated in both ‘Harlikar’/‘M9-Nic29’ and ‘Harlikar’/‘71-3-150’. Multiple genes encoding typhasterol/6-deoxotyphasterol 2alpha-hydroxylase [*MD09G1181200*, *MD02G1092100*, *MD02G1093200* (*CYP92A6*)] were up-regulated in ‘Harlikar’/‘M9-Nic29’ and ‘Harlikar’/‘71-3-150’ (Figure 9B).

The cytochrome P450 monooxygenase (P450) gene *MD10G1138400* (*CYP87A3*), involved in BR biosynthesis, was significantly up-regulated in both ‘Harlikar’/‘M9-Nic29’ and ‘Harlikar’/‘71-3-150’ but down-regulated in ‘Harlikar’/‘Qingzhen 2’ (Figure 9C).

Most of the 38 DEGs involved in the biosynthesis and signal transduction of ABA were up-regulated in ‘Harlikar’/‘M9-Nic29’ and ‘Harlikar’/‘71-3-150’, while ABA responsive element binding factor *MD11G1163900* (*ABF*) in ‘Harlikar’/‘Qingzhen 2’ were down-regulated (Figure 9D).

## 3. Discussion

Although grafting is widely used in apple production, some common rootstocks still exhibit graft incompatibility in orchards as scion varieties are updated and iterated [32]. The rational use of rootstocks can improve tree structure, enhance stress resistance, regulate the production period, and improve fruit quality [33]. Kumar et al. [34] reported that the growth vigor of trees grafted onto ‘EMLA111’ and ‘EMLA7’ was larger than those on ‘BUD9’. Li et al. [30] found that ‘Mac9’, as a dwarfing interstock, could significantly reduce the height and diameters of ‘Gala’/‘*M. baccata*’ apple trees, while having few effects on the growth of annual branches. Chen et al. [16] reported that ‘Fuji’ apple trees grafted onto ‘*M. baccata*’ presented a larger branch architecture than ‘Fuji’/‘M9-T337’ and ‘Fuji’/‘M9-T337’/‘*M. baccata*’. Lordan et al. [35] found ‘Honeycrisp’/‘B72020’ was the stock with more upright branches, while ‘Honeycrisp’/‘B10’ and ‘Honeycrisp’/‘G11’ had the flattest. ‘*M. baccata*’ is a traditional vigorous rootstock widely used in China [36]. Nowadays, dwarfism is one of main objectives for rootstock selecting and breeding [31].

To select suitable dwarfing rootstocks for ‘Harlikar’, a widely cultivated apple variety in China in recent years, we grafted ‘Harlikar’ scions onto seven dwarfing rootstocks (Figure 2). ‘M9’ is the most commonly used dwarfing rootstock in the world because of its suitability for high-density plantings [37], and many excellent lines selected from ‘M9’ as well as its hybrid posterity, such as ‘M9-T337’, ‘M9-Nic29’, and ‘M9-Pajam 2’ and ‘M26’ have been widely used for crop production in some countries and regions [38]. ‘M9-T337’ confers early fruiting and high yields [39]. The rooting and branching abilities of ‘M9-Nic29’ were better than ‘M9’ [40]. ‘M9-Pajam 2’ is the most vigorous subclone of ‘M9’, from a range of tested types, and it also has better rooting and branching abilities than ‘M9’ [40,41,42]. Gjamovski et al. [41] reported that ‘M9-T337’ with less vigorous was suitable for high-density apple orchards, while ‘M9-Pajam 2’ was recommended for less vigorous apple cultivars and rare apple plantations. The semi-dwarfing rootstock ‘M26’ shows good stationarity and grafting affinity, and has stronger cold resistance compared with ‘M7’, ‘JM7’, ‘M9-T337’ and ‘M9’ [43]. ‘B9’ can offer several advantages, including strong tree architecture, uniform in fruiting, best in quality, and resistance to pests, diseases, and cold [44], and it is shown to be more dwarfing than M9 series [45]. According to the research of Yin et al. [46], the semi-lethal temperature of ‘71-3-150’ reached −40.63 °C, its cold resistance was better than ‘GM256’ and ‘M9’. ‘Qingzhen 2’ is semi-dwarfing apomixis rootstock with a large open branching angle, deep root distribution and wide-ranging adaptability, and suitable for most small-scale orchards in China with poor soil conditions [47]. In this study, among eight apple scion/rootstock combinations used in this study, the highest survival rate of scions were observed in ‘Harlikar’/‘Qingzhen 2’ (Figure 1), however, its dwarfism was not prominent (Figure 3). Besides, the survival rate of ‘Harlikar’ grafted onto M series such as ‘M9-T337’, ‘M9-Nic29’ and ‘M9-Pajam2’ was higher than that of ‘Harlikar’ grafted onto ‘*M. baccata*’. And the affinity of ‘Harlikar’ with cold-resistant apple dwarfing rootstocks ‘B9’ and ‘71-3-150’ was superior to that of ‘Harlikar’ with ‘*M. baccata*’. The above-ground material accumulation in the ‘Harlikar’/‘B9’, ‘Harlikar’/‘M9-T337’, ‘Harlikar’/‘M9-Nic29’, ‘Harlikar’/‘M9-Pajam2’ and ‘Harlikar’/‘71-3-150’ combinations was lower than in ‘Harlikar’/‘*M. baccata*’ (Figure 3E). And the above five rootstocks could be used as candidate dwarfing rootstocks for ‘Harlikar’, ‘71-3-150’ could be prioritized in north China, where the climate is cold and dry in winter [48].

Our results showed that ‘Harlikar’ was less compatible with ‘M26’ (Table A1). Graft compatibility/incompatibility is a complex response that includes a wide range of anatomical, physiological, and biochemical interactions. Successful grafting begins with an initial wound. Tissues from the scion cultivar are fused with the rootstock to form one composite organism [49]. At the graft interface of ‘Harlikar’/‘M26’, there was less callus formed (Figure A1E). Even though the leaf buds of scions sprouted in the early stage of grafting, the survival rate was lower than that of the other seven scion/rootstock combinations over time (Figure 1). Foliage etiolation is an observed symptom of graft incompatibility [32]. For example, ‘Hongmian miyou’ is mutated from ‘Guanxi miyou’, but these two scions show different compatibility with available ‘*Poncirus trifoliata*’ rootstock, and phloem collapse with cell wall distortion and thickening in the prominent midribs, as well as foliage etiolation were much more frequently observed in ‘Hongmian miyou’/‘*P. trifoliata*’ [32]. Chen et al. [49] reported that a compatible combination had higher chlorophyll content and net photosynthetic rate than an incompatible combination in litchi. Consistent with the results from the present study, the *P_n_*, *G_s_*, *T_r_*, and SPAD of ‘Harlikar’ grafted onto ‘M26’ was lowest (Figure 4A,B,D,E), and foliage etiolation was obvious (Figure 2E). The SPAD index values indicated restricted carbohydrate assimilation and N uptake due to translocated incompatibility [5]. Therefore, ‘M26’ is not suitable for ‘Harlikar’ in north China.

Apart from ‘Harlikar’/‘M26’, the height of ‘Harlikar’ grafted onto ‘71-3-150’ was lowest in this study (Figure 3). Differently, ‘Harlikar’/‘71-3-150’ had the highest leaf SPAD values among the eight apple scion/rootstock combinations (Figure 4E, Table A3), and foliage etiolation was not observed in the leaves of scions (Figure 2). Thus, ‘71-3-150’ may post positive effects on the dwarfing of ‘Harlikar’, the molecular biology of the dwarfing mechanism should be further clarified. Additionally, we found that the number of lateral branches in ‘Harlikar’/‘Qingzhen 2’ was higher than that in the other seven scion/rootstock combinations (Table A1), and the branch opening angle in ‘Harlikar’/‘Qingzhen 2’ was close to 90 degrees (Figure 2G). Therefore, ‘Qingzhen 2’ had remarkable effects on the tree architecture of ‘Harlikar’, and the mechanism needs to be further investigated.

The key to rootstock-scion interaction is recognition and information flow between the rootstock and scion cells. Phloem tubes contain hundreds of types of RNA, including short RNAs, noncoding RNAs and mRNAs. These RNAs can affect various aspects of plant growth, such as leaf development, fruit quality, and root formation [30]. Therefore, we hope to screen key genes regulating dwarfing traits, and branching architecture in apple using transcriptome technology. Tree vigor is mainly controlled by hormone responses and nutrient availability [22]. Hormonal regulation has been proposed as an important mechanism through which rootstocks influence scion morphology by changing shoot-root chemical signaling [24]. Li et al. [14] suggested that the oxidation or degradation of auxin in the phloem may lead to dwarfing of apple rootstocks. In this study, auxin-repressed protein gene *ARP* (*MD05G1311800*) was up-regulated in ‘Harlikar’/‘71-3-150’ and ‘Harlikar’/‘M9-Nic29’ (Figure 9A). It was closely related to the dwarfing trait of ‘Harlikar’/‘71-3-150’ and ‘Harlikar’/‘M9-Nic29’. The levels of endogenous CKs in plants are regulated by isopentenyl transferases (IPT), cytochrome P450 monooxygenase (family 735, subfamily A), CK oxidase/dehydrogenase (CKX) and other biosynthetic and degrading enzymes. The tZ-type CKs are biosynthesized via CYP735A and have a specific function in shoot growth promotion [50]. Kiba et al. [51] demonstrated that *CYP735A3* and *CYP735A4* encode cytochrome P450 monooxygenase (P450), required for tZ-type side-chain modification in rice, which function in promoting shoot and root growth. tZ-type CK biosynthesis is negatively regulated by auxin, ABA, and CK, and positively regulated by dual N nutrient signals, namely glutamine-related and nitrate-specific signals. In this study, *CKXs* were down-regulated in ‘Harlikar’/‘M9-Nic29’ and ‘Harlikar’/‘71-3-150’, and *CYP735A* was down-regulated in ‘Harlikar’/‘M9-Nic29’ (Figure 9B). The down-regulation of these genes may lead to decreased CK contents, which are associated with lower plant height in the two rootstock/scion combinations. Sun et al. [52] isolated *ZmCYP90D1*, which encodes a member of the cytochrome P450 family, from a spontaneous dwarfing mutation of maize, *m30*, with decreased internode number and length but increased internode diameter. *ZmCYP90D1* could influence plant height and stalk diameter via hormone-mediated cell division and cell growth via the BR pathway. The differences in height among ‘Harlikar’/‘M9-Nic29’, ‘Harlikar’/‘71-3-150’, and ‘Harlikar’/‘Qingzhen 2’ may be due to the transcription levels of *CYP87A3* and *CYP92A6*, which involved in BR biosynthesis in apple (Figure 9C). In addition, studies have shown that plant height is closely related to the synthesis of GAs. Zhang et al. [25] noted the up-regulation of the gibberellin 3β-hydroxylase gene *GA3ox* and the accumulation of GAs in dwarfed mangoes, while Hooijdonk et al. [15] suggested that disrupting GA metabolism plays a vital role in dwarfing the scion. Li et al. [30] found that auxin and GA were inhibited, and abscisic acid was promoted in apple trees when using dwarfing interstocks for grafting [5]. BR promotes plant cell elongation through a complex signaling cascade that modulates the activities of growth-related genes and through interaction with GAs [53,54]. In addition, multiple GA-responsive genes, such as *GASAs* (*MD15G1246100*, *MD08G1088900*, *MD14G1081900*, and *MD02G1132400*) and *LOB* (*MD09G1088700*), were down-regulated (Figure A4A). Hayat et al. [24] reported high expression of *GID1*, a GA receptor gene, in the leaves of dwarfing rootstock, suggesting that it might reduce the biological activity of GA. Consistent results were also found in this study. *GID1* (*MD12G1227200*) was markedly down-regulated in ‘Harlikar’/‘Qingzhen 2’ leaves but up-regulated in ‘Harlikar’/‘M9-Nic29’ leaves (Figure A4A). Phenotypically, ‘Harlikar’/‘M9-Nic29’ displayed obvious dwarfing characteristics compared with ‘Harlikar’/‘Qingzhen 2’. The interaction of BR and GA at the signaling and metabolic levels has been confirmed to influence cell elongation and plant height control in rice and Arabidopsis [53]. Therefore, it can be inferred that BR-GA interaction affected plant height in different apple rootstock/scion combinations. Furthermore, the abscisic-aldehyde oxidase gene *AAO3* (*MD11G1178400*), the gene for an enzyme that catalyzes the final steps of ABA synthesis (9-cis-epoxycarotenoid dioxygenase) *NCED* (*MD10G1194200*), and multiple ABA-response-related genes were significantly up-regulated. These DEGs were closely related to the dwarfing trait of ‘Harlikar’/‘71-3-150’ (Figure 9D).

Phytohormones are known to influence branching. CKs promote shoot branching by activating axillary buds. There is a strong specialization involving the ARR-independent mechanism of CK signaling that controls shoot branching, in which type-A ARR proteins are negative regulators of transcriptional CK signaling (type-A mutants exhibit reduced bud activation), whereas type-B ARR proteins positively regulate CK-induced gene expression [55]. In this study, ARR-A family member *MD14G1188400* was up-regulated in ‘Harlikar’/‘Qingzhen 2’ (Figure 9C), and it may be one of the reasons for the higher number of lateral branches in ‘Harlikar’/‘Qingzhen 2’ (Figure 2 and Figure 3C, Table A1). Eduardo et al. [56] reported that TCP transcription factor BRANCHED1 (BRC1) binds to and positively regulates the transcription of three related Homeodomain leucine zipper protein-encoding genes, enhancing *NCED3* expression and promoting ABA accumulation, thereby suppressing bud development. Moreover, ABA-responsive binding factors (ABF) was induced by ABA [57]. Zhu et al. [58] found that GA positively regulates the branching angle of tea plants, the cytokinin metabolism genes *CKX* and the abscisic acid signal transduction gene *PYL4* were involved in the formation of branching angles in tea plants. Consistent results were found in this study, as the ABA-responsive element binding factor gene *ABF* (*MD11G1163900*) was down-regulated in ‘Harlikar’/‘Qingzhen 2’, while it was significantly up-regulated in ‘Harlikar’/‘M9-Nic29’. In addition, *NCED1* (*MD10G1194200*) and *AAO3* (*MD11G1178400*) were up-regulated in ‘Harlikar’/‘71-3-150’ (Figure 9D). The down-regulation of *ABF* may be the another key reason to increased branching in ‘Harlikar’/‘Qingzhen 2’ (Figure 2G, Table A1). Furthermore, it this study, *CKX* (*MD15G1021500*) and *PYL4* (*MD07G1227100*, *MD12G1178800*, and *MD04G1165000*) were down-regulated in ‘Harlikar’/‘M9-Nic29’ and ‘Harlikar’/‘71-3-150’, but not down-regulated expressed in ‘Harlikar’/‘Qingzhen 2’ compared with ‘Harlikar’/‘*M. baccata*’ (Figure 9B,D). These genes may be closely related to the branching angle formation in apple.

**Figure A5:**
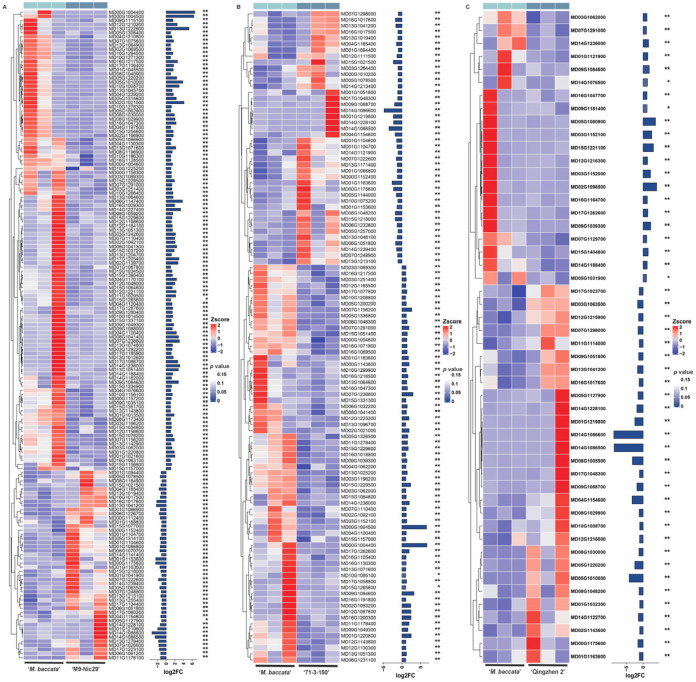
Heatmap of differentially expressed genes (DEGs) related to nitrogen in ‘Harlikar’ grafted onto three different rootstocks. The DEGs in comparisons of (**A**) ‘Harlikar’/‘M9-Nic29’ vs. ‘Harlikar’/‘*M. baccata*’, (**B**) ‘Harlikar’/‘71-3-150’ vs. ‘Harlikar’/‘*M. baccata*’, and (**C**) ‘Harlikar’/‘Qingzhen 2’ vs. ‘Harlikar’/‘*M. baccata*’. The colors in the heatmap represent FPKM values after Z-score processing, with blue indicating higher expression and red indicating lower expression. * represents a significant difference in gene expression between different scion/rootstock combinations (*p* < 0.05), ** represents a highly significant difference (*p* < 0.01).

**Figure A6:**
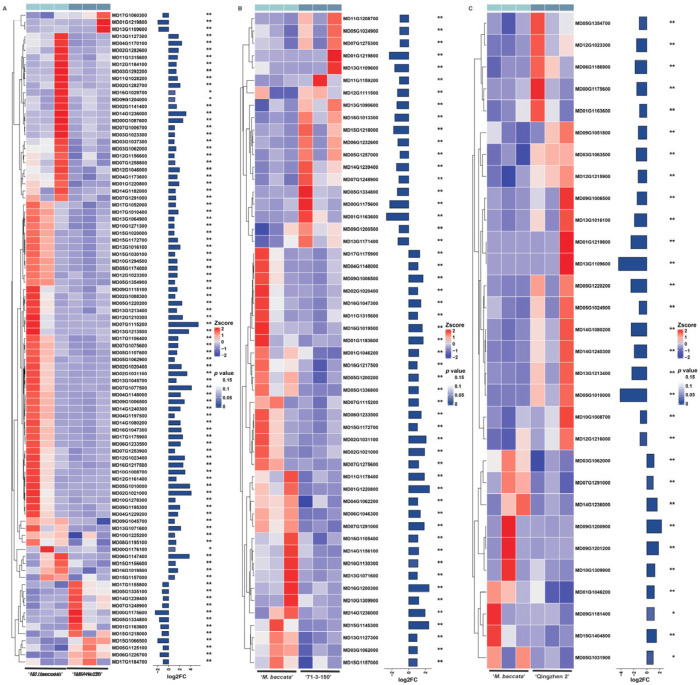
Heatmap of differentially expressed genes (DEGs) related to phosphorus in ‘Harlikar’ grafted onto three different rootstocks. The DEGs in comparisons of (**A**) ‘Harlikar’/‘M9-Nic29’ vs. ‘Harlikar’/‘*M. baccata*’, (**B**) ‘Harlikar’/‘71-3-150’ vs. ‘Harlikar’/‘*M. baccata*’, and (**C**) ‘Harlikar’/‘Qingzhen 2’ vs. ‘Harlikar’/‘*M. baccata*’. The colors in the heatmap represent FPKM values after Z-score processing, with blue indicating higher expression and red indicating lower expression. * represents a significant difference in gene expression between different scion/rootstock combinations (*p* < 0.05), ** represents a highly significant difference (*p* < 0.01).

**Figure A7:**
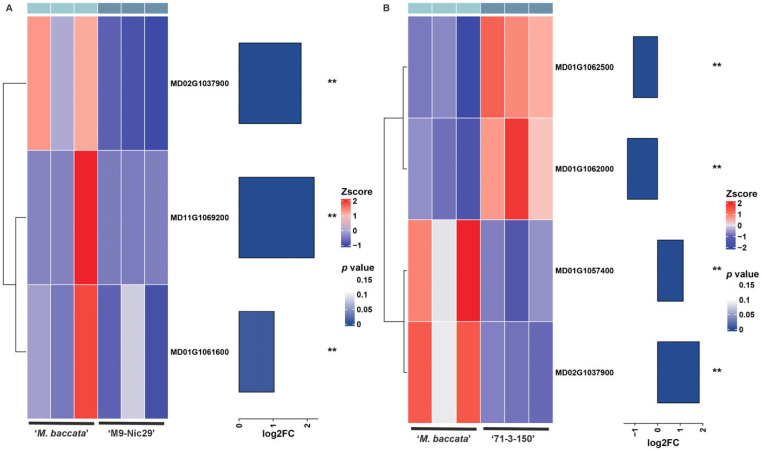
Heatmap of differentially expressed genes (DEGs) related to potassium in ‘Harlikar’ grafted onto three different rootstocks. The DEGs in comparisons of (**A**) ‘Harlikar’/‘M9-Nic29’ vs. ‘Harlikar’/‘*M. baccata*’, (**B**) ‘Harlikar’/‘71-3-150’ vs. ‘Harlikar’/‘*M. baccata*’. The colors in the heatmap represent FPKM values after Z-score processing, with blue indicating higher expression and red indicating lower expression. ** represents a highly significant difference (*p* < 0.01).

We also noted that, although there was no difference in total N accumulation between the roots of ‘*M. baccata*’ and ‘Qingzhen 2’, the total N accumulation in leaves and stems of ‘Harlikar’ grafted onto ‘Qingzhen 2’ was significantly higher than that of the scion grafted onto ‘*M. baccata*’ (Figure 5A, Table A4). Moreover, the total P and K accumulation in the roots of ‘Harlikar’/‘*M. baccata*’ was higher than in ‘Harlikar’/‘Qingzhen 2’, but that in the leaves and stems of scions grafted onto ‘*M. baccata*’ was significantly lower than in ‘Harlikar’/‘Qingzhen 2’ (Figure 5A, Table A5 and Table A6). Furthermore, ‘Harlikar’/‘Qingzhen 2’ grew taller than ‘Harlikar’/‘*M. baccata*’ (Figure 3A). We speculate that there were some DEGs related to nutrient utilization between ‘Harlikar’/‘Qingzhen 2’ and ‘Harlikar’/‘*M. baccata*’, which affected the growth of scions. The availability of nutrients, particularly N and P, is crucial for plant development, and grafting affects the movement of these substances through the xylem [59]. The DEGs screened from three scion/rootstock combinations were enriched in cellular acid phosphatase activity (GO:0004674) (Figure A2). Liu et al. [60] found that acid phosphatase (coded for by *ACP2*) primarily catalyzed the hydrolysis of phosphorethanolamine and phospho-L-serine, and elevated serine levels reduced the expression of genes involved in the photorespiratory pathway of an *ACP2*-overexpression line. Our findings revealed the down-regulation of several acid phosphatase genes *ACPs* (*MD13G1016100*, *MD12G1023300*, and *MD14G1240300*) and serine/threonine-protein kinase genes *PBLs* (*MD05G1010000*, *MD05G1220200*, and *MD10G1008700*) in ‘Harlikar’/‘Qingzhen 2’. In contrast, these genes were all up-regulated in ‘Harlikar’/‘M9-Nic29’ (Figure A5 and Figure A6). The up-regulation of *ACPs* and serine/threonine-protein kinase genes in ‘Harlikar’/‘M9-Nic29’ may inhibit photosynthesis, hindering the vegetative growth of scions. On the contrary, the *P_n_* of leaves in ‘Harlikar’/‘Qingzhen 2’ significantly increased, thus enhancing the aboveground growth (Figure 3A and Figure 4A). Compared with ‘Harlikar’/‘*M. baccata*’, the potassium channel protein gene (*MD02G1037900*) *AKT1* and multiple receptor-like protein genes such as *EIX1s (MD01G1062000, MD01G1062500)* and *EIX2 (MD01G1061600)* were significantly up-regulated in the leaves of ‘Harlikar’/‘M9-Nic29’ and ‘Harlikar’/‘71-3-150’, and the potassium transporter gene *HAK1* (*MD11G1069200*) was up-regulated in the leaves of ‘Harlikar’/‘M9-Nic29’ (Figure A7). Therefore, the total K contents in the above-ground parts of ‘Harlikar’/‘M9-Nic29’ and ‘Harlikar’/‘71-3-150’ were significantly higher than in ‘Harlikar’/‘*M. baccata*’ (Figure 5C). Compared with ‘Harlikar’/‘*M. baccata*’, the aquaporin gene *MD01G1121900* (*TIP1*) was significantly up-regulated in the leaves of ‘Harlikar’/‘Qingzhen 2’ (Figure A5). Water-N coupling positively affects the yield, quality, water- and N-use efficiencies of crops [61]. The up-regulation of *TIP1* may, therefore, improve the absorption of water and nutrients in ‘Harlikar’/‘Qingzhen 2’. In brief, multiple genes related to hormone signaling and nutrient utilization were involved in the regulation process of rootstock on the growth, development, and formation of lateral branches of apple scion.

## 4. Materials and Methods

### 4.1. Plant Materials and Growth Conditions

Eight scion/rootstock combinations were used in this study. The scion was ‘Harlikar’, an excellent apple variety selected by Professor Kiyoshi Yokota from the natural hybrid offspring of Golden Crown at Iwate University’s Department of Agriculture [29]. The three-year-old apple rootstocks included seven dwarfing rootstocks, ‘M9-T337’, ‘M9-Nic29’, ‘M9-Pajam2’, ‘M26’, ‘B9’, ‘71-3-150’, and ‘Qingzhen 2’ and a vigorous rootstock ‘*M. baccata*’. The virus-free superior line ‘M9-T337’ was selected from ‘M9’ by the Dutch General Inspection of Woody Seedlings [62]. ‘M9-Nic29’ was selected from ‘M9’ by Rene Nicolai Nursery in Belgium [62]. The patented rootstocks ‘M9-Pajam 2’ was selected from ‘M9’ by the French CTIFL [42]. The semi-dwarfing rootstock ‘M26’, selected from ‘M16’ × ‘M9’ as the parent at the East Molin Experimental Station in the UK [63]. ‘B9’ was a dwarfing rootstock selected from a cross between ‘M8’ and ‘Krasniy Shtandart’ [64]. ‘71-3-150’ was selected from a cross between ‘Budagovsky’s Paradise’ and ‘58-257’ [65]. ‘Qingzhen 2’ is a new semi-dwarfing apomixis apple rootstock obtained from gamma-ray mutagenesis of ‘*Malus hupehensis* var. mengshanensis’ stratified seeds by Qingdao Academy of Agricultural Sciences, China, offspring have high uniformity [47]. After rooting, the tissue-cultured ‘*M. baccata*’ were used in this study to ensure that the genetic background of the material was consistent.

In the autumn of 2019, the rootstock plants were planted in a gallon basin (36 cm top diameter, 32 cm bottom diameter, 29 cm high). The cultivation substrate was washed river sand (25 kg). The apple rootstocks were placed in the rain shelter of the experimental orchard at the Chinese Academy of Agricultural Sciences (CAAS), Xingcheng, China (120°440 E, 40°370 N), and regularly irrigated with modified 1/2 Hoagland nutrient solution to ensure the proper growth. In May 2022, 30 healthy and budded plants were selected from each type of rootstock, and healthy and full ‘Harlikar’ leaf buds were grafted onto the rootstock trunks at 10 cm above the ground. Each rootstock was grafted separately with one scion leaf bud. The excess rootstock trunk was cut off after the scion was established. Routine management was carried out to ensure that the other experimental conditions were consistent.

### 4.2. Sample Collection and Determination

#### 4.2.1. Measurements of Growth Parameters

In July 2022, the number of elongated scion leaf buds was counted, and the survival rate was calculated as the ratio of the number of elongated scion leaf buds to the total number of grafted scion leaf buds. Subsequently, on 2 July 2022, the photosynthetic parameters of the fully expanded leaves on the elongated scions were measured using an LI-6400 Portable Photosynthesis System (LI-COR 6400XT; LI-COR, Lincoln, NE, USA), 5–7 mature functional leaves were selected from each plant, the central part of the leaf without vein was used for determination. The photosynthetic parameters were determined for six times for each plant, and three plants for each scion/rootstock combination. And the chlorophyll content was measured using a SPAD-502 chlorophyll meter (Konica Minolta, Inc., Tokyo, Japan). In October 2022, plant height and internode length were measured with a tape measure (±0.01 cm), the number of nodes was counted, and the stem diameters of the scion were measured at 5 cm above the graft union with Vernier calipers (±0.01 mm). Then, the roots, stems, and leaves of three experimental replications from each combination were harvested and the fresh weights measured.

#### 4.2.2. Determination of Mineral Concentration

After weighing, the roots, stems, and leaves of three experimental replications from each combination were washed three times with distilled water, fixed at 105 °C for 30 min, and dried at 70 °C to a constant weight before being ground into a powder. Then, 0.3 g of powdered material from each tissue type was digested using H_2_SO_4_-H_2_O_2_. The total P content was determined using the vanadium molybdate yellow colorimetric method. The total N content was determined using the Kjeldahl method. The total K content was determined using a flame spectrophotometer.

#### 4.2.3. RNA Extraction, Library Construction, and Transcriptome Sequencing

Four different cDNA libraries were constructed using scion leaves from ‘Harlikar’/‘M9-Nic29’, ‘Harlikar’/‘71-3-150’, ‘Harlikar’/‘Qingzhen 2’, and ‘Harlikar’/‘*M. baccata*’ for transcriptomic profiling. Three biological replicates were assessed for each treatment. Total RNA was extracted using the RNAprep Pure Plant kit (DP441, Tiangen, Beijing, China). The high-quality mRNA was randomly fragmented. cDNA libraries were obtained using the Hieff NGS^®^ Ultima Dual-mode mRNA Library Prep Kit (Yeasen Biotechnology Co., Ltd., Shanghai, China; 12309ES96) and sequenced using a Novaseq6000 system (Illumina, San Diego, CA, USA).

#### 4.2.4. Bioinformatics Analysis

Raw reads were filtered to remove low-quality tags, empty tags, and tags with only one copy number. Clean reads were mapped to the reference sequences (Malus x domestica GDDH13 v1.1 Whole Genome Assembly & Annotation, https://www.rosaceae.org/species/malus/malus_x_domestica/genome_GDDH13_v1.1, accessed on 5 September 2024) using the STAR Maximal Mappable Prefix (MMP) method. Gene expression levels were determined using the FPKM method. Differential expression analysis was performed using DESeq2 with a design formula [|log2 (FoldChange)| ≥ 1 and false discovery rate (FDR) < 0.05] that took into account the contrast among samples from four scion/rootstock combinations. The genes with an adjusted *p*-value of ≤0.05 using multi-testing error correction (Benjamini-Hochberg test) and corresponding to FDR of 1%, were considered DEGs.

#### 4.2.5. Statistical Analysis and Visualization

All physiological data were graphed using GraphPad Prism v6.01 (GraphPad Software Inc., Boston, MA, USA). Duncan’s test (*p* < 0.05) was used to analyze the statistical significance by SAS v9.0 (SAS Institute Inc., Cary, NC, USA).

## 5. Conclusions

In conclusion, seven rootstock including ‘M9-T337’, ‘M9-Nic29’, ‘M9-Pajam2’, ‘B9’, and ‘71-3-150’ could be used as candidate dwarfing rootstocks for ‘Harlikar’. There were significant differences in plant height and branching traits among different rootstock/scion combinations. ‘Harlikar’ with ‘71-3-150’ as the rootstock had stronger dwarfing traits, while ‘Qingzhen 2’ as the rootstock could have excellent branching ability. This study explored the formation mechanism underlying apple scion architecture, and identified the key factors affecting plant height and lateral branch development. Through physiological and transcriptomic analyses, key genes involved in dwarfing and branching development were identified. Genes related to hormone synthesis, metabolism, and signal transduction, as well as nutrient absorption and utilization, participate in this complex regulatory process in apple.

## Data Availability

The data that support the findings of this study are available from the corresponding author upon reasonable request.

## Abbreviations

The following abbreviations are used in this manuscript:

N	Nitrogen
P	Phosphorus
K	Potassium
ABA	Abscisic acid
CK	Cytokinin
BR	Brassinosteroid
GA	Gibberellin

## Appendix A

**Table A1:** The raw data of plant height, scion stem diameter, scion node number, and scion internode length of ‘Harlikar’ apple with different rootstocks.

Rootstocks	Height (cm)	Stem Diameter (mm)	Internode Length (cm)	Node Number	Branch Number
B9	54.67 ± 0.98 b	5.410 ± 0.253 cd	25.07 ± 0.17 a	23.00 ± 2.91 ab	0 ± 0 b
M9-T337	56.17 ± 5.20 b	5.753 ± 0.509 c	24.08 ± 0.49 a	24.67 ± 3.76 b	0 ± 0 b
M9-Nic29	52.67 ± 1.36 b	5.263 ± 0.093 cd	20.43 ± 0.12 a	24.33 ± 2.22 b	0 ± 0 b
M9-Pajam2	55.00 ± 0.82 b	5.748 ± 0.113 c	25.41 ± 0.09 a	24.00 ± 1.24 ab	0 ± 0 b
M26	24.83 ± 2.11 d	3.347 ± 0.427 e	22.83 ± 0.41 b	13.33 ± 1.24 b	0 ± 0 b
71-3-150	36.17 ± 1.77 c	4.512 ± 0.234 de	22.84 ± 0.18 b	16.00 ± 2.09 b	2.3 ± 0.7 b
Qingzhen 2	71.67 ± 0.72 a	9.108 ± 0.205 a	33.63 ± 0.30 a	26.33 ± 1.58 a	10.0 ± 3.0 a
*M. baccata*	64.00 ± 1.96 b	7.287 ± 0.333 b	25.83 ± 1.11 a	25.83 ± 1.11 ab	5.0 ± 0.6 ab

Note: Data were shown as means ± SE (*n* = 3). Different lowercase letters after the data in the same column in the table indicated significant difference between different apple scion/rootstock combinations (*p* < 0.05).

**Table A2:** The raw data of fresh weight of ‘Harlikar’ apple with different rootstocks.

Rootstocks	Leaves	Stems	Roots
B9	13.29 ± 0.41 bc	9.08 ± 0.62 cd	31.38 ± 2.51 b
M9-T337	13.13 ± 1.83 bc	10.48 ± 2.11 c	18.15 ± 3.70 b
M9-Nic29	11.97 ± 0.80 cd	8.85 ± 0.23 cd	23.46 ± 3.21 b
M9-Pajam2	15.98 ± 1.02 bc	10.56 ± 0.65 c	28.58 ± 0.95 b
M26	3.08 ± 0.69 e	2.06 ± 0.51 e	14.59 ± 3.30 b
71-3-150	7.28 ± 0.80 de	4.81 ± 0.53 de	25.20 ± 3.13 b
Qingzhen 2	39.90 ± 3.26 a	30.41 ± 2.22 a	204.75 ± 27.68 a
*M. baccata*	28.98 ± 1.01 b	21.79 ± 0.94 b	225.11 ± 7.48 a

Note: Data were shown as means ± SE (*n* = 3). Different lowercase letters after the data in the same column in the table indicated significant difference between different apple scion/rootstock combinations (*p* < 0.05).

**Table A3:** The raw data of photosynthetic parameters of scion leaves in ‘Harlikar’ apple with different rootstocks.

Rootstocks	*P_n_*	*G_s_*	*C_i_*	*T_r_*	SPAD
B9	19.10 ± 0.34 bc	0.193 ± 0.006 b	242.33 ± 8.46 a	2.38 ± 0.02 ab	36.53 ± 0.69 ab
M9-T337	17.50 ± 0.37 c	0.185 ± 0.005 b	237.83 ± 6.34 a	1.28 ± 0.05 d	35.28 ± 1.37 abc
M9-Nic29	19.37 ± 0.28 b	0.214 ± 0.008 b	263.33 ± 4.75 a	1.91 ± 0.21 c	32.48 ± 0.45 bcd
M9-Pajam2	21.83 ± 0.10 a	0.153 ± 0.015 b	171.00 ± 25.15 a	1.21 ± 0.10 d	37.13 ± 0.16 a
M26	1.39 ± 0.56 d	0.007 ± 0.001 c	185.68 ± 75.17 a	0.12 ± 0.01 e	29.39 ± 2.03 d
71-3-150	17.77 ± 0.59 bc	0.191 ± 0.022 b	253.00 ± 13.59 a	2.10 ± 0.15 bc	38.60 ± 1.79 a
Qingzhen 2	22.40 ± 0.59 a	0.281 ± 0.024 a	274.00 ± 14.51 a	2.50 ± 0.06 a	32.07 ± 0.36 bcd
*M. baccata*	20.50 ± 0.23 bc	0.227 ± 0.022 b	265.00 ± 22.76 a	1.80 ± 0.05 d	30.99 ± 0.77 cd

Note: Data were shown as means ± SE (*n* = 3). Different lowercase letters after the data in the same column in the table indicated significant difference between different apple scion/rootstock combinations (*p* < 0.05).

**Table A4:** The raw data of total nitrogen content in ‘Harlikar’ apple with different rootstocks.

Rootstocks	Leaves	Stems	Roots
B9	81.78 ± 10.71 bc	25.85 ± 3.02 cd	62.74 ± 10.94 b
M9-T337	126.73 ± 19.25 b	32.53 ± 8.81 bc	28.13 ± 6.26 b
M9-Nic29	33.89 ± 1.33 cd	18.68 ± 0.94 cd	43.07 ± 8.20 b
M9-Pajam2	112.95 ± 24.57 b	26.43 ± 2.66 cd	55.71 ± 1.45 b
M26	14.23 ± 4.60 d	4.95 ± 2.13 e	42.51 ± 13.56 b
71-3-150	40.01 ± 5.25 cd	11.07 ± 1.35 de	40.62 ± 8.98 b
Qingzhen 2	193.56 ± 43.07 a	145.51 ± 11.11 a	546.79 ± 115.31 a
*M. baccata*	107.16 ± 7.49 b	45.72 ± 6.10 b	529.75 ± 31.19 a

Note: Data were shown as means ± SE (*n* = 3). Different lowercase letters after the data in the same column in the table indicated significant difference between different apple scion/rootstock combinations (*p* < 0.05).

**Table A5:** The raw data of total phosphorus content in ‘Harlikar’ apple with different rootstocks.

Rootstocks	Leaves	Stems	Roots
B9	20.94 ± 0.93 bc	16.70 ± 2.73 b	38.43 ± 3.10 c
M9-T337	20.58 ± 2.24 bc	15.40 ± 4.47 bc	20.16 ± 4.78 c
M9-Nic29	17.58± 0.33 bc	9.22 ± 0.27 bcd	21.27 ± 4.27 c
M9-Pajam2	23.69± 2.40 b	8.01 ± 0.19 cd	39.06 ± 3.03 c
M26	3.22± 1.02 d	1.80 ± 0.71 d	19.78 ± 5.42 c
71-3-150	11.59± 0.88 cd	5.49 ± 0.58 d	25.66 ± 4.97 c
Qingzhen 2	56.65± 8.44 a	30.25 ± 4.59 a	214.55 ± 26.23 b
*M. baccata*	25.05± 1.83 b	16.42 ± 1.88 a	314.97 ± 32.47 a

Note: Data were shown as means ± SE (*n* = 3). Different lowercase letters after the data in the same column in the table indicated significant difference between different apple scion/rootstock combinations (*p* < 0.05).

**Table A6:** The raw data of total potassium content in ‘Harlikar’ apple with different rootstocks.

Rootstocks	Leaves	Stems	Roots
B9	351.88 ± 21.33 b	156.62 ± 16.82 b	182.45 ± 12.14 c
M9-T337	319.33 ± 38.33 b	177.78 ± 38.77 b	110.58 ± 33.93 c
M9-Nic29	339.32 ± 12.83 b	166.86 ± 8.36 b	130.01 ± 29.53 c
M9-Pajam2	332.27 ± 29.75 b	174.93 ± 10.83 b	157.98 ± 19.32 c
M26	49.76 ± 11.29 d	25.02 ± 9.19 d	83.51 ± 43.30 c
71-3-150	224.88 ± 9.67 bc	87.17 ± 13.86 cd	128.92 ± 17.10 c
Qingzhen 2	1257.40 ± 140.56 a	543.53 ± 49.66 a	1648.29 ± 284.38 b
*M. baccata*	91.20 ± 5.17 cd	61.43 ± 3.15 d	2183.33 ± 158.69 a

Note: Data were shown as means ± SE (*n* = 3). Different lowercase letters after the data in the same column in the table indicated significant difference between different apple scion/rootstock combinations (*p* < 0.05).

**Table A7:** Summary of RNA sequencing data from leaves of ‘Harlikar’ grafted with ‘71-3-150’, ‘M9-Nic29’, ‘Qingzhen 2’, and ‘*M. baccata*’.

Sample	Raw Reads	Raw Bases	Clean Reads	Clean Bases	Clean GC Content	Filter Ratio
71-3-150_1	46.624760 M	6.993714 G	45.708520 M	6.806628 G	46.9497	0.980349
71-3-150_2	48.604918 M	7.290738 G	47.778752 M	7.103569 G	46.98	0.983002
71-3-150_3	51.496616 M	7.724492 G	50.691978 M	7.557973 G	46.883	0.984375
M9-Nic29_1	37.310086 M	5.596513 G	36.595928 M	5.446360 G	47.1604	0.980859
M9-Nic29_2	36.038730 M	5.405810 G	35.286442 M	5.259532 G	47.3643	0.979126
M9-Nic29_3	48.192588 M	7.228888 G	47.363384 M	7.052710 G	46.9985	0.982794
*M. baccata*_1	46.229260 M	6.934389 G	45.409060 M	6.763222 G	47.0605	0.982258
*M. baccata*_2	36.844798 M	5.526720 G	36.107638 M	5.380199 G	46.9463	0.979993
*M. baccata*_3	42.811104 M	6.421666 G	41.973686 M	6.251795 G	47.1838	0.980439
Qingzhen 2_1	46.948802 M	7.042320 G	46.151644 M	6.873791 G	47.1655	0.983021
Qingzhen 2_2	39.988378 M	5.998257 G	39.222534 M	5.842204 G	47.0958	0.980848
Qingzhen 2_3	40.953742 M	6.143061 G	40.138188 M	5.975946 G	47.0593	0.980086
